# AIDS-Associated Cryptosporidial and Cytomegalovirus Cholangiopathy

**DOI:** 10.7759/cureus.63963

**Published:** 2024-07-06

**Authors:** Nada M Alsharif, Mamoun M Souleiman, Luxhman Gunaseelan, Cecilia Big

**Affiliations:** 1 Internal Medicine, Corewell Health Dearborn Hospital, Dearborn, USA; 2 Infectious Disease, Corewell Health Dearborn Hospital, Dearborn, USA

**Keywords:** cytomegalovirus, hiv aids, cholecystitis, cholangiopathy, cryptosporidium infection

## Abstract

Acquired immune deficiency syndrome (AIDS)-associated cholangiopathy is a biliary tract condition seen in AIDS patients who are severely immunosuppressed, contributing to significant mortality in this population, even in developed countries with access to highly active antiretroviral therapy (HAART).

We discuss a thirty-six-year-old human immunodeficiency virus (HIV)-positive male, non-compliant with HAART therapy, who presented with a one-year history of weight loss, persistent fatigue, and chronic diarrhea, which had worsened significantly in the past few weeks. Routine laboratory studies on presentation indicated elevated liver enzymes and alkaline phosphatase, a CD4 count of 2 cells/mm^3^, and a high HIV RNA count of 8.8 million. Imaging via CT of the abdomen and pelvis and ultrasound of the abdomen both displayed thickening and edema in the gallbladder without evidence of gallstones, raising concerns of acalculous cholecystitis. The patient subsequently decompensated, requiring intravenous vasopressors to maintain hemodynamic stability, broad-spectrum antibiotics, and resumption of antiretroviral therapy. Biliary fluid drainage was performed, and Cryptosporidium and cytomegalovirus (CMV) were detected via polymerase chain reaction (PCR) testing. The diagnosis of AIDS cholangiopathy was established; however, the patient's diarrhea worsened upon the introduction of tube feeds. Despite ongoing antimicrobial treatment, the patient developed a fever of 101.4°F, became asystolic and subsequently passed away. This case highlights the diagnostic, management, and therapeutic challenges of AIDS cholangiopathy. Also, it underscores the importance of thorough investigation into even mild or intermittent diarrhea and abnormal liver function tests in all HIV-infected patients, particularly in severely immunosuppressed patients.

AIDS cholangiopathy should be considered in AIDS patients with diarrhea and abnormal liver function tests, irrespective of age, due to its associated morbidity across all age groups. Laboratory investigations often reveal markedly elevated alkaline phosphatase, gamma-glutamyltransferase, and mild to moderate liver enzyme elevations as hallmark findings of AIDS cholangiopathy. Ultrasonography is the first-line screening modality of AIDS cholangiopathy. *Cryptosporidium parvum* is the most common infectious etiology of AIDS cholangiopathy and can be identified by DNA-based polymerase chain reaction (PCR) testing of the stool or biliary fluid or acid-fast staining of stool specimens.

Early detection of HIV infection and the prompt initiation and adherence to highly active antiretroviral therapy (HAART), which helps with maintaining a normal CD4 count and a low HIV viral load through HAART therapy, thereby significantly reducing the risk of developing AIDS cholangiopathy in HIV patients.

## Introduction

Cholangiopathies have been observed among individuals with acquired immune deficiency syndrome (AIDS). These conditions were initially identified in human immunodeficiency virus (HIV) patients during the 1980s [1], predominantly affecting men who have sex with men, with a mean age of 37 years [2]. However, the incidence of AIDS-related cholangiopathy has notably declined since the mid-1990s with the introduction of antiretroviral therapy [3]. Occurrences are now primarily confined to patients with limited access to antiretroviral treatments, those who do not comply with medication regimens, particularly those with severely low CD4 counts vulnerable to opportunistic infections, and individuals with drug-resistant strains of the human immunodeficiency virus (HIV) [4].

Here, we present a case that showcases a relatively rare manifestation in an HIV-positive patient who was ultimately diagnosed with AIDS-associated cryptosporidial cholangiopathy. This case highlights our experience in managing such a patient and outlines the outcome of the treatment.

## Case presentation

A 36-year-old male presented to the emergency department with a one-year history of persistent fatigue and chronic diarrhea, which had worsened significantly in the prior few weeks. During a review of his symptoms, a weight loss of 15 pounds over the preceding six months was noted. The patient denied experiencing nausea, vomiting, or abdominal pain. He was diagnosed with HIV in 2006 through routine screening. Although he has been prescribed bictegravir, emtricitabine, and tenofovir alafenamide in recent years, due to concerns about potential renal toxicity, he was non-adherent to the prescribed regimen.

**Figure 1:**
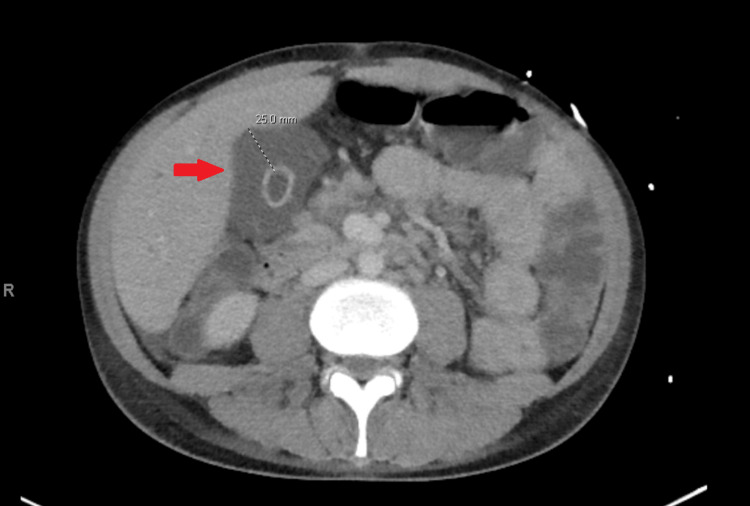
CT abdomen with contrast shows significant gallbladder mucosal thickening and edema. Gallbladder wall measures 25 mm in thickness (red arrow), without evidence of cholelithiasis.

**Figure 2:**
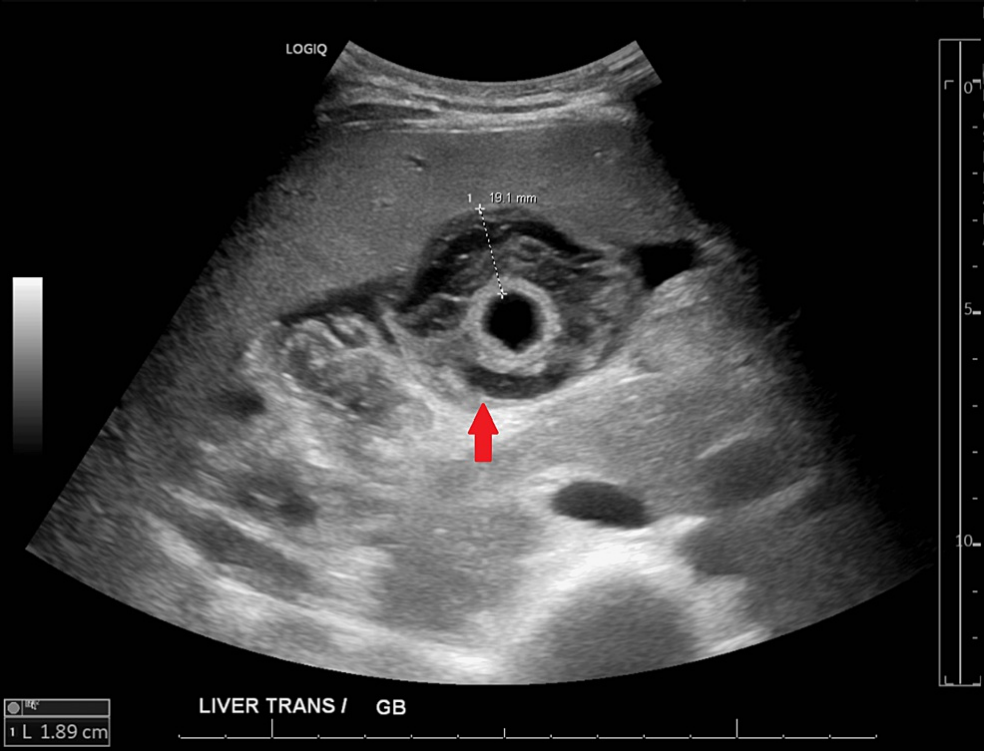
Ultrasound abdomen shows significant gallbladder mucosal thickening and edema. Gallbladder wall measures 19.1 mm in thickness (red arrow). There is no evidence of cholelithiasis.

Upon admission, the patient exhibited a fever of 100.4°F, hypotension with a blood pressure of 85/42, and a rapid heart rate of 127 beats per minute. Physical examination revealed tenderness in the right upper quadrant. Laboratory studies on presentation indicated elevated levels of alkaline phosphatase (ALP) (1048), aspartate aminotransferase (AST) (118), and alanine aminotransferase (ALT) (129), significantly higher than his baseline values of ALP (331), AST (68), and ALT (116) (Table 1). His CD4 count in the emergency department was alarmingly low at 2, with a high HIV RNA count of 8.8 million. Serology for hepatitis B and C came back negative. Imaging via CT abdomen and pelvis (Figure 1) displayed thickening and edema in the gallbladder without evidence of gallstones, a finding confirmed by abdominal ultrasound (Figure 2), raising concerns of acute or chronic acalculous cholecystitis.

**Table 1:** Lab values on presentation. Please note the elevated ALP, transaminases, and direct hyperbilirubinemia signifying the cholestatic pattern of liver injury. Also, note the low CD4 count and high HIV RNA count are suggestive of AIDS. ALP: alkaline phosphatase, AST: aspartate aminotransferase, ALT: alanine aminotransferase, HIV: human immunodeficiency virus, AIDS: acquired immune deficiency syndrome.

Laboratory test	Result	Normal range
ALP	1048	33-120 u/L
AST	118	<35 U/L
ALT	129	9-47 U/L
Bilirubin, total	1.8	0.3-1.2 mg/dL
Bilirubin, direct	1.5	0.0-0.4 mg/dL
CD4 count	2	433-1722 mil/L
HIV RNA count	8.8 million	Not applicable
Hepatitis B serology	Negative	Negative
Hepatitis C serology	Negative	Negative

Despite initial volume resuscitation, the patient's hypotension persisted, necessitating the use of intravenous vasopressors. He received broad-spectrum antimicrobials, including IV ceftriaxone, IV metronidazole, and trimethoprim-sulfamethoxazole for *Pneumocystis pneumonia* (PCP) and Toxoplasma prophylaxis. His self-discontinued HIV antiretroviral therapy (bictegravir, emtricitabine, and tenofovir alafenamide) was resumed. Interventional radiology placed a cholecystostomy tube. A subsequent stool analysis revealed a positive result for Cryptosporidium antigen, prompting the initiation of nitazoxanide three days after admission. Blood and urine cultures and serum tests for fungal infections, including Fungitell and galactomannan, yielded negative results.

**Figure 3:**
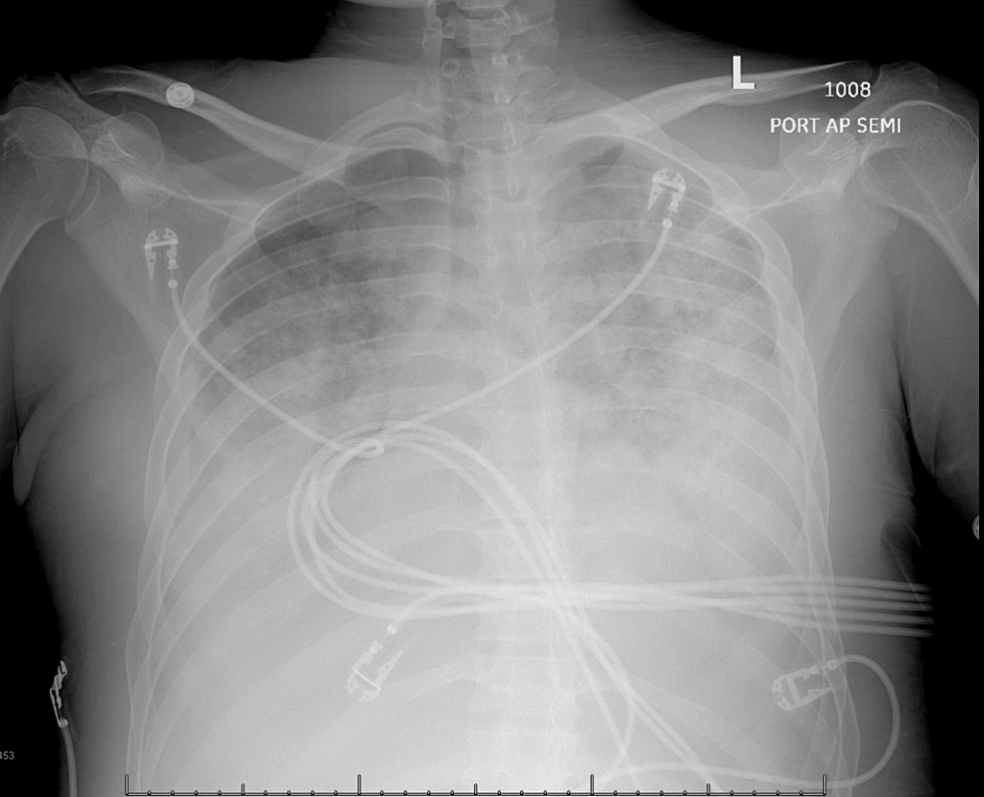
Chest X-ray shows bilateral patchy opacities with pulmonary vascular congestion.

During the hospitalization, the patient's kidney function deteriorated, leading to anuria. Given his unstable hemodynamics, continuous renal replacement therapy (CRRT) was initiated. Although the requirement for intravenous vasopressors decreased, the patient developed worsening hypoxia, necessitating intubation. A chest X-ray (Figure 3) revealed bilateral patchy opacities with pulmonary vascular congestion, attributed to volume overload.

The patient's diarrhea initially improved with nitazoxanide therapy but worsened upon the introduction of tube feeds. Biliary fluid analysis confirmed cytomegalovirus (CMV) via PCR testing, and the modified Kinyoun stain from concentrated bile was also positive for *Cryptosporidium parvum*. Therefore, he was started on ganciclovir while continuing nitazoxanide. Despite ongoing antimicrobial treatment, the diarrhea persisted, and the patient developed a fever of 101.4°F. Later that day, the patient experienced asystole, prompting cardiopulmonary resuscitation (CPR). Return of spontaneous circulation (ROSC) was achieved after four rounds of CPR. Subsequently, the patient's family elected to change his code status to do not resuscitate (DNR), and the patient passed away on the same day.

## Discussion

AIDS cholangiopathy, recognized as a well-documented biliary syndrome prevalent in individuals with AIDS or severe immunocompromise, arises due to the formation of strictures in the biliary tract prompted by opportunistic infections. These strictures lead to biliary obstruction and subsequent cholestatic liver damage [5].

Clinical manifestations of AIDS cholangiopathy often present in severely immunocompromised patients, featuring persistent right upper quadrant pain coupled with symptoms like nausea, vomiting, and diarrhea. The intensity of pain can vary, contingent upon the existence of papillary stenosis. Additionally, jaundice may appear, indicating potential biliary obstruction [2]. Patients commonly exhibit a low-grade fever, as observed in our case initially, yet may experience high-grade fever spikes if bacterial cholangitis ensues. Considerable weight loss, as seen in our patient, is another prevalent symptom. Physical examinations typically reveal abdominal tenderness and hepatomegaly, consistent with this patient's findings [2].

Laboratory investigations often reveal markedly elevated alkaline phosphatase, gamma-glutamyltransferase, and mild to moderate liver enzyme elevations as hallmark findings of AIDS cholangiopathy. Notably, our patient displayed a significant elevation in alkaline phosphatase disproportionate to the elevation in liver enzymes. It's worth noting that in about one-fourth of patients, AIDS cholangiopathy may present subtly without biochemical abnormalities, despite evident cholangiography findings [6]. 

Ultrasonography is a useful initial screening modality of AIDS cholangiopathy, as it can identify the intraluminal caliber and thickness of the bile ducts, the thickness of the gallbladder wall, the presence of stones or pericholecystic fluid [7]. Endoscopic ultrasound is preferred compared to transabdominal ultrasound due to the better detection of dilation and thickening of the common bile duct and the exclusion of stones, extra biliary compression, and tumors. CT imaging is used to identify intrahepatic strictures associated with AIDS cholangiopathy and other hepatic and pancreatic abnormalities [8]. 

As 20% of patients exhibit normal noninvasive imaging studies, diagnosing AIDS cholangiopathy typically requires direct visualization of the biliary tracts through endoscopic retrograde cholangiopancreatography (ERCP) [9]. Biliary brushings or aspirated biliary fluid can be evaluated for Cryptosporidium or Microsporidia, and biopsy samples can identify CMV, Cryptosporidium, and Mycoplasma. Additionally, patients experiencing severe pain can undergo sphincterotomy and stent placement for relief. ERCP, however, is not recommended for patients with asymptomatic AIDS cholangiopathy [10]. AIDS cholangiopathy is associated with the following findings on ERCP: intrahepatic sclerosing cholangitis-like lesions (20%), papillary stenosis (15%), a combination of the above (50%), and long extrahepatic bile duct strictures (15%). 

Infectious disease workups, including stool and biliary fluid analysis, typically identify opportunistic pathogens, notably Cryptosporidium. *Cryptosporidium parvum* is the most common pathogen associated with AIDS cholangiopathy and has been isolated in up to 57% of patients [4]. Cryptosporidium appears to synergize with HIV in the biliary system, inducing apoptosis in infected cholangiocytes through the fast/fast ligand system, a process further potentiated by the HIV-1 trans-activator of transcription (TAT) protein [11]. *Cryptosporidium parvum *also contributes to autonomic nerve damage in the intestines, resulting in sphincter of Oddi dysfunction, papillary stenosis, and subsequent biliary tract dysmotility [12]. Intraluminal polypoid defects are also seen in the common bile duct or near intrahepatic strictures. The combination of papillary stenosis and intrahepatic ductal strictures is unique to AIDS cholangiopathy [12]. 

Other pathogens commonly associated with AIDS cholangiopathy include Mycobacterium complex (MAC) infections, Microsporidia and *Enterocytozoon bieneusi*, and rarely Isospora [13]. Mycobacterium complex (MAC) infections are associated with granulomatous biliary tract obstruction, resulting in biliary obstruction findings similar to Cryptosporidium infections [6]. Viruses, such as cytomegalovirus, are also commonly associated with AIDS cholangiopathy, as in the case of our patient [13]. 

Sometimes, no pathogens are identified when evaluating for AIDS cholangiopathy [14]. In such situations, consider malignancies such as Kaposi's sarcoma and Burkitt's lymphoma, as they can also lead to AIDS cholangiopathy [15]. However, many of the pathogens that are associated with AIDS cholangiopathies, such as *Cryptosporidium parvum*, Microsporidia, and *Enterocytozoon bieneusi*, are difficult to detect, sometimes requiring multiple biopsies and special stains [13]. Also, testing for Cryptosporidium and other pathogens is typically limited to stool samples in many hospital systems, therefore contributing to the diagnostic challenge. In our case, PCR testing for Cryptosporidium from the bile sample was done off-label at our request, and the results could not be officially reported by our lab. 

This case emphasizes the importance of considering the risk of Cryptosporidium cholangitis in AIDS patients with diarrhea and abnormal liver function tests, irrespective of age, due to the potential morbidity and mortality associated with Cryptosporidium infection across all age groups. Furthermore, for Cryptosporidium detection, utilizing immunofluorescent-based procedures and PCR proves more effective than stool microscopy, particularly during symptomatic periods. For instance, our patient's Cryptosporidium infection was identified promptly in stool via antigen detection and much later via off-label PCR testing of the bile.

Additionally, early detection of HIV infection and the prompt initiation and adherence to highly active antiretroviral therapy (HAART) play a crucial role. Maintaining a normal CD4 count and a low HIV viral load through HAART therapy can significantly reduce the likelihood of developing AIDS cholangiopathy in HIV patients [3].

## Conclusions

In summary, our case highlights a fatal case of AIDS cholangiopathy triggered by a cryptosporidial and CMV infection that surfaced at a relatively young age. Our patient initially presented with seemingly benign symptoms of intermittent diarrhea and abnormal liver function tests, yet rapidly progressed to a state of decompensation. This case highlights the diagnostic, management, and therapeutic challenges of AIDS cholangiopathy. Also, it underscores the importance of thorough investigation into even mild or intermittent diarrhea and abnormal liver function tests in all HIV-infected patients, especially during the severely immunosuppressed stages. Early initiation and consistent adherence to HAART significantly influence and improve the potential for a favorable outcome in such cases.
